# Totally Endoscopic Aortic Valve Replacement Using a Longitudinal Incision for a Type 0 Bicuspid Aortic Valve: A Case Report

**DOI:** 10.7759/cureus.93050

**Published:** 2025-09-23

**Authors:** Hitoki Hashiguchi, Naomi Yasuda, Akihito Ohkawa, Kyousuke Miki

**Affiliations:** 1 Cardiovascular Surgery, Hokkaido Prefectural Kitami Hospital, Kitami, JPN

**Keywords:** bicuspid aortic valve, hybrid suture technique, longitudinal incision, minimally invasive cardiac surgery, paravalvular leak prevention, totally endoscopic aortic valve replacement, type 0 bav

## Abstract

A 70-year-old man with severe aortic stenosis due to type 0 bicuspid aortic valve (BAV) (New York Heart Association (NYHA) class II) and mild aortic regurgitation (AR) underwent totally endoscopic aortic valve replacement (AVR) using a longitudinal aortotomy. Preoperative coronary angiography showed no obstructive coronary artery disease. A 29-mm bioprosthesis (INSPIRIS RESILIA, Edwards Lifesciences, Irvine, CA, USA) was implanted with a hybrid suture strategy (everting mattress at the nadirs plus interrupted sutures elsewhere). The longitudinal incision provided direct annular exposure, enabling implantation of a large prosthesis and facilitating annular circularization. Postoperative transthoracic echocardiography demonstrated an aortic valve area of 2.32 cm² with a mean pressure gradient of 6 mmHg and a maximum gradient of 11 mmHg, and no residual aortic regurgitation (no paravalvular or transvalvular components); physiologic intra-prosthetic washout jets may be present but are not classified as AR. Computed tomography on postoperative day (POD) 6 confirmed circular remodeling of the previously elliptical annulus. Recovery was uneventful, and the patient was discharged on POD 7. This case highlights the feasibility of an exposure-driven longitudinal aortotomy for totally endoscopic AVR in type 0 BAV and its potential role in annular remodeling; long-term durability requires further study.

## Introduction

Bicuspid aortic valve (BAV) is the most common congenital valvular anomaly, associated with complex anatomy, asymmetrical calcifications, and challenging surgical management [1].

Although the Sievers and Schmidtke classification has been the most widely adopted system for BAV morphology [1], an international consensus classification was recently published in 2021, which incorporates not only the number of raphes but also valve phenotype, cusp symmetry, valve function, and associated aortopathy [2]. This case involved a Sievers type 0 BAV, consistent with the consensus classification of a symmetric, raphe-less BAV with an elliptical annulus.

Among its variants, type 0 BAV, characterized by the absence of a raphe (a fibrous ridge formed by fused leaflets) and often large, elliptical annuli, presents particular difficulties for both surgical and transcatheter approaches [3,4]. These anatomical features can predispose to paravalvular leakage (PVL) and suboptimal valve expansion, especially in transcatheter aortic valve implantation (TAVI) [5,6].

The longitudinal incision is a recently developed vertical aortotomy designed specifically for totally endoscopic aortic valve replacement (TE-AVR). Unlike conventional transverse or oblique aortotomies, it provides a direct vertical incision toward the non-coronary sinus, allowing improved endoscopic visualization and exposure of the annulus. This facilitates precise suturing and implantation of large prosthetic valves. The technique may be particularly advantageous in cases with complex annular anatomy such as type 0 BAV. To our knowledge, reports of TE-AVR utilizing a longitudinal incision in type 0 BAV are limited.

The objective of this report is to present a case of severe aortic stenosis in a patient with type 0 BAV successfully managed with longitudinal incision TE-AVR, and to discuss the rationale, surgical details, and outcomes.

## Case presentation

A 70‑year‑old man with a remote history of asymptomatic aortic regurgitation (AR) diagnosed in his 30s was referred for evaluation of a newly detected systolic murmur and electrocardiographic abnormalities. He had no significant comorbidities or prior surgical history.

On admission, his vital signs were stable (temperature 36.5°C, blood pressure 132/56 mmHg, heart rate 58 bpm, SpO₂ 98%), and his functional status corresponded to New York Heart Association (NYHA) class II.

Preoperative testing

**Figure 1:**
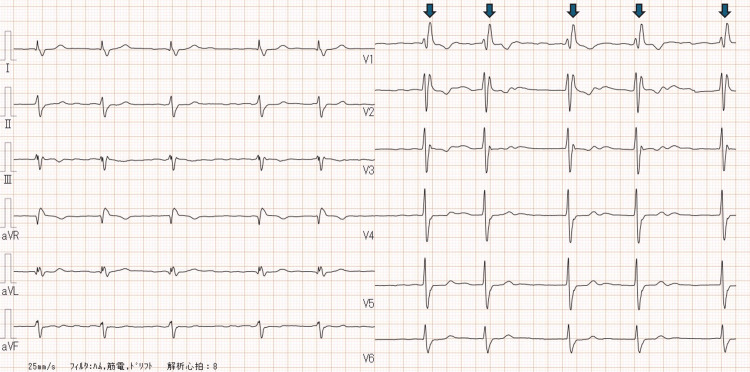
Preoperative electrocardiogram (ECG) The 12-lead ECG shows atrial fibrillation, as evidenced by an irregular RR interval (arrows) and the absence of distinct P waves. A complete right bundle branch block (RBBB) pattern is also present, characterized by a widened QRS complex and terminal R waves in leads V1–V2 with wide, slurred S waves in leads I, V5–V6.

**Figure 2:**
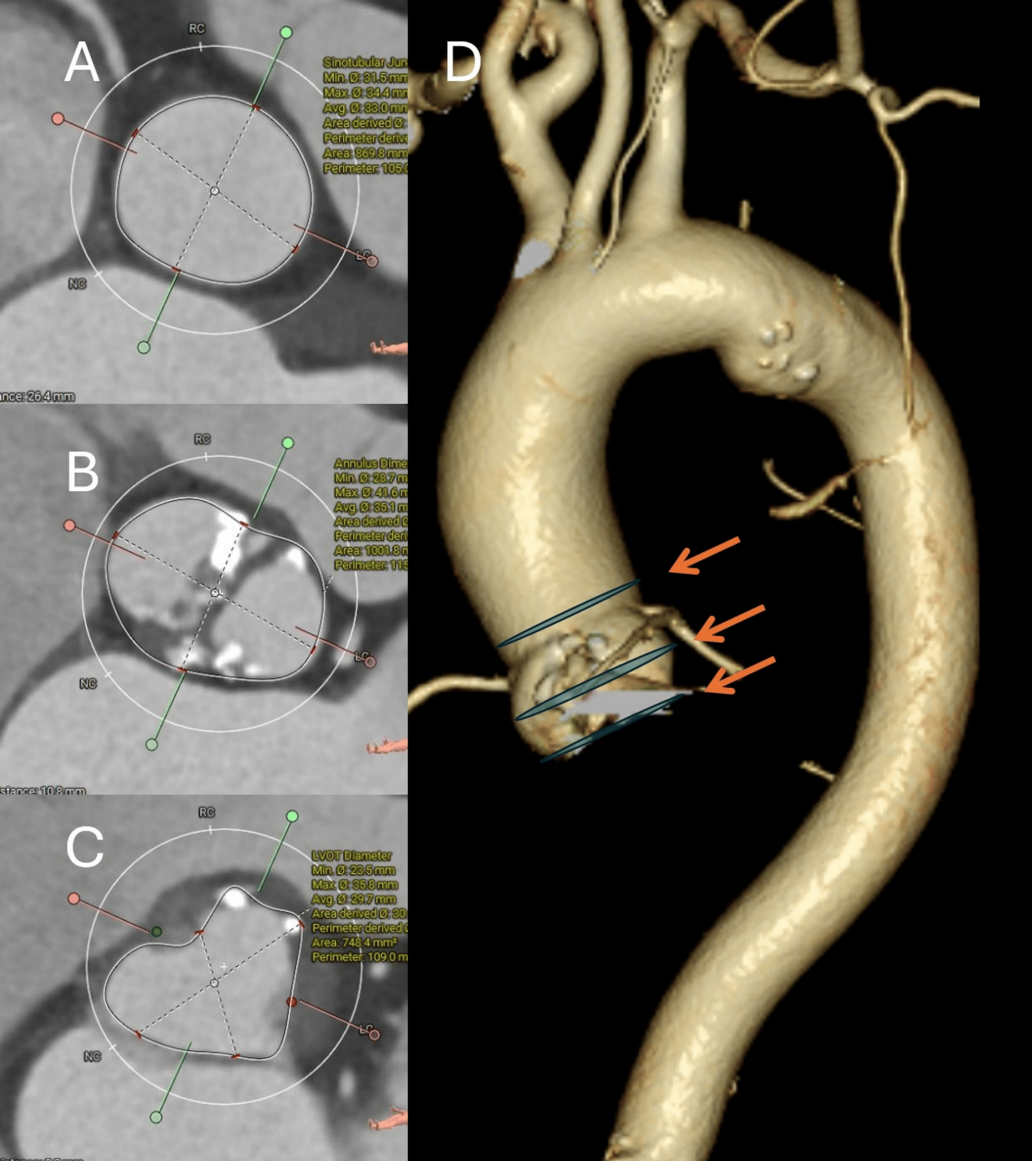
Preoperative computed tomography (CT) images. (A) Cross-sectional view at the sinotubular junction (STJ). (B) Cross-sectional view at the sinuses of Valsalva (SOVs). (C) Cross-sectional view at the left ventricular outflow tract (LVOT). (D) Three-dimensional reconstructed CT image of the aorta. The measured diameters were as follows: Ascending aorta 40×37 mm, STJ 39×29 mm, SOVs 41×28 mm, annulus 39×27 mm, LVOT 41×25 mm, femoral artery (FA) and external iliac artery (EIA) both < 9 mm.

The 12‑lead electrocardiogram (ECG) demonstrated atrial fibrillation with a complete right bundle branch block pattern (Figure 1). Transthoracic echocardiography (TTE) confirmed severe aortic stenosis with a maximum velocity of 5.0 m/s, a mean pressure gradient of 55 mmHg, a calculated aortic valve area of 0.70 cm², and a preserved left ventricular ejection fraction of 61%. Mild concomitant AR was present. Computed tomography (CT) showed an elliptical annulus and the following diameters (Figure 2): ascending aorta 40×37 mm, sinotubular junction (STJ) 39×29 mm, sinuses of Valsalva (SOVs) 41×28 mm, annulus 39×27 mm, and left ventricular outflow tract (LVOT) 41×25 mm. The femoral artery (FA) and external iliac artery (EIA) were both < 9 mm in diameter. Preoperative coronary angiography (CAG) demonstrated no obstructive coronary artery disease.

Given the patient’s type 0 BAV anatomy, the risk of elliptical expansion and paravalvular leakage with TAVI was considered high. Standard open AVR was an alternative, but the patient strongly preferred a minimally invasive option. TE-AVR using a longitudinal incision was selected as the most suitable approach to achieve optimal annular exposure and implantation of a large prosthesis.

Operative findings and technique

**Figure 3:**
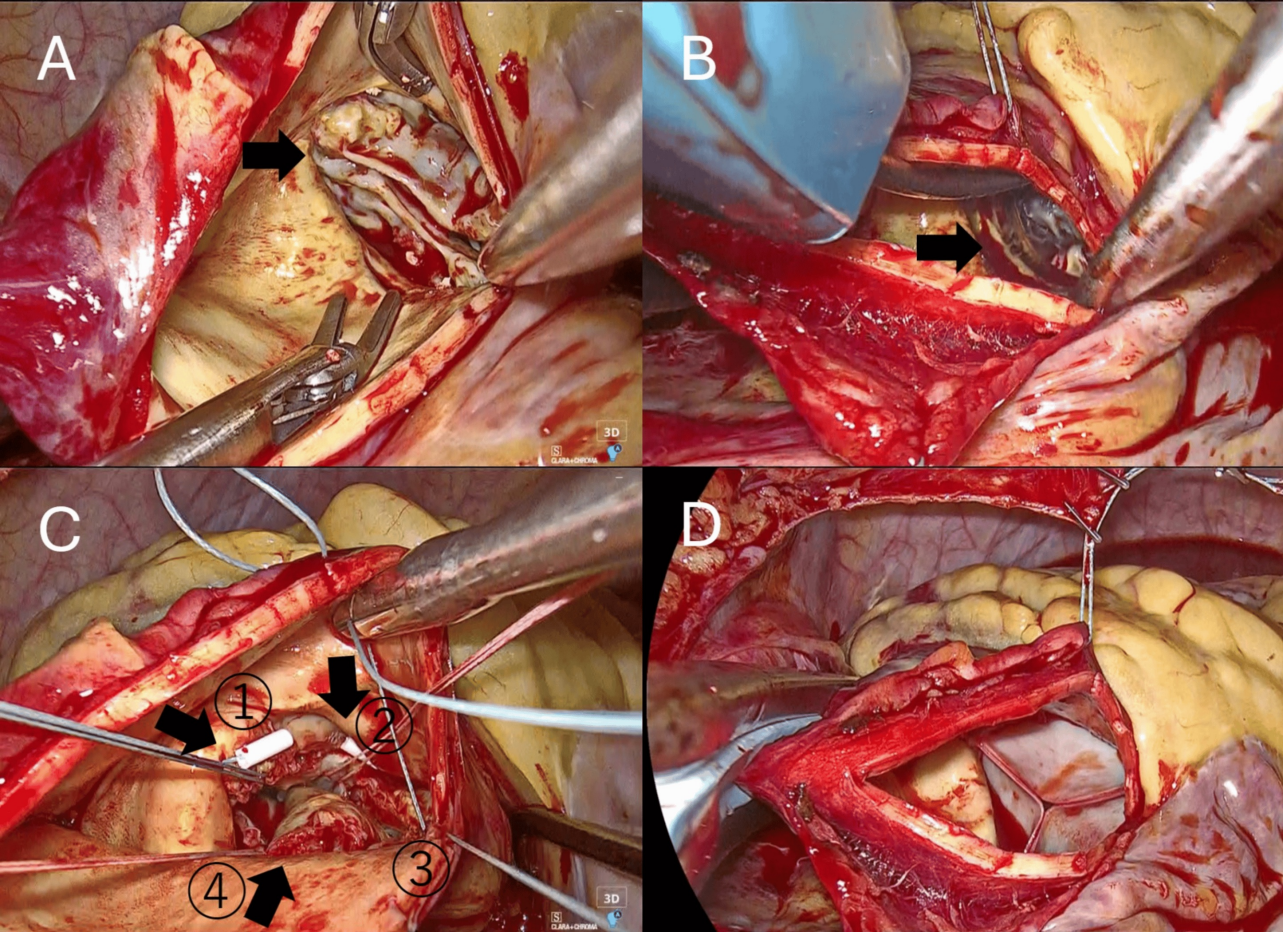
Intraoperative endoscopic views. (A) Exposure of the aortic valve using a longitudinal incision, revealing a type 0 bicuspid aortic valve. (B) Sizing of the annulus with a 29-mm INSPIRIS sizer. (C) Placement of four everting mattress sutures at the nadirs and commissures. (D) Final view after implantation of a 29-mm INSPIRIS valve.

Through a totally endoscopic 4K 3D approach, a longitudinal aortotomy approximately 4 cm in length was created in the ascending aorta, oriented vertically toward the non‑coronary sinus and stopping about 1 cm proximal to the STJ. This incision afforded direct endoscopic visualization of a Sievers type 0 bicuspid aortic valve with an elliptical annulus (Figure 3A). After complete excision of the native cusps, four everting mattress sutures with pledgets were placed at the nadirs and commissures (clock positions 0, 3, 6, and 9 o’clock), and interrupted sutures were placed at the remaining eight positions to balance annular tension (hybrid suture technique). The annulus was sized with a 29‑mm INSPIRIS RESILIA sizer (Edwards Lifesciences, Irvine, CA, USA) (Figure 3B), and a 29‑mm INSPIRIS bioprosthesis was implanted in the intra‑annular position without difficulty (Figure 3D). No intraoperative complications occurred. An edited intraoperative video illustrating the exposure and suturing sequence is provided (Video 1).

**Video 1:** Totally endscopic aortic valve replacement＋left atrial appendage closure for Type 0 bicuspid aortic valve Totally endoscopic view demonstrating the longitudinal aortotomy incision and hybrid suture technique for implantation of a 29-mm INSPIRIS bioprosthesis in a patient with Type 0 bicuspid aortic valve. The video also shows subsequent left atrial appendage closure performed using a Penditure 45 mm device.

Postoperative course and imaging

**Figure 4:**
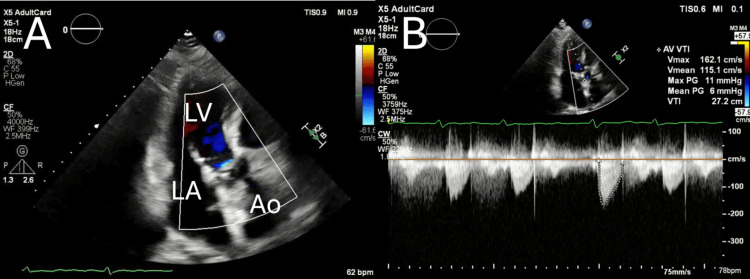
Postoperative transthoracic echocardiography (TTE). (A) Color Doppler imaging demonstrates only trivial transvalvular leakage following valve implantation. (B) Continuous-wave Doppler across the aortic valve shows a peak velocity in the 1 m/s range, with a maximum pressure gradient of 11 mmHg and a mean pressure gradient of 6 mmHg.

**Figure 5:**
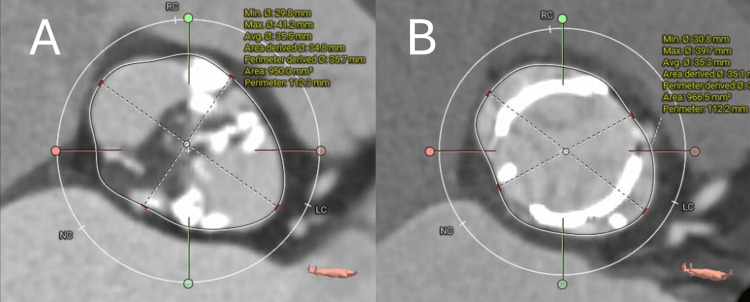
Pre- and postoperative CT curved planar reconstruction (CPR) images at the annular level. (A) Preoperative image shows an elliptical-shaped annulus characteristic of Type 0 bicuspid aortic valve. (B) Postoperative image demonstrates a more circular expansion of the annulus achieved with the hybrid suture technique, addressing the anatomical challenge that predisposes to paravalvular leakage.

The patient was extubated on POD 1, transferred from the high‑care unit on POD 2, and ambulated with >4 metabolic equivalents (METs) prior to discharge on POD 7. Postoperative TTE demonstrated an aortic valve area of 2.32 cm², a maximum pressure gradient of 11 mmHg, and a mean pressure gradient of 6 mmHg, with no residual aortic regurgitation (no paravalvular or transvalvular AR); physiologic intra‑prosthetic washout jets typical of bioprostheses may be present but are not classified as AR (Figure 4, Table 1). CT obtained on POD 6 showed remodeling of the annulus from an elliptical to a more circular configuration (Figure 5).

**Table 1:** Summary of key echocardiographic parameters (pre- vs post-operative) Physiologic intra-prosthetic washout jets typical of bioprostheses may be present but are not categorized as AR. PVL: paravalvular leakage

Parameter	Pre-operative	Post-operative
Peak aortic velocity (Vmax)	5.0 m/s	1.6 m/s
Maximum pressure gradient	98 mmHg	11 mmHg
Mean pressure gradient	55 mmHg	6 mmHg
Aortic valve area (AVA)	0.70 cm²	2.32 cm²
Left ventricular ejection fraction (LVEF)	61%	66%
Aortic regurgitation (AR)	Mild	None *(no PVL or transvalvular AR)*

## Discussion

Type 0 BAV presents unique challenges due to its elliptical annulus and the absence of a raphe, which complicates both TAVI and sutureless valve implantation [3-6]. In TAVI, the prosthesis often expands elliptically, resulting in residual gradients and a higher incidence of aortic regurgitation [5-7]. Standard surgical AVR allows leaflet excision and circular prosthetic expansion, but minimally invasive exposure can be limited, particularly in totally endoscopic approaches.

The longitudinal incision enhances annular exposure by providing a direct vertical line of sight to the valve, enabling accurate annular sizing and secure placement of a large prosthesis. In this case, the technique, combined with a hybrid suture strategy, achieved effective circular remodeling, no residual aortic regurgitation, and excellent hemodynamic results. Compared to conventional endoscopic aortotomy, the longitudinal incision offers superior visualization and may reduce the risk of paravalvular leakage in anatomically challenging BAV cases. In this case, no residual aortic regurgitation was observed postoperatively (no paravalvular or transvalvular components); physiologic intra‑prosthetic washout jets may be seen but are not categorized as AR, confirming the adequacy of circular remodeling.

A concise comparison of BAV classification systems (Sievers and Schmidtke vs. the 2021 international consensus) is provided in Table 2 [1,2].

**Table 2:** Morphological classification systems for bicuspid aortic valve (BAV) AS: aortic stenosis, AR: aortic regurgitation

Classification system	Main criteria	Subtypes / Features	Notes
Sievers & Schmidtke (2007)	Based on number of raphes	- Type 0: No raphe- Type 1: One raphe- Type 2: Two raphes	Most widely used; simple and practical
International Consensus (2021)	Integrative classification including:- Valve phenotype & symmetry- Raphe (presence, number, calcification)- Cusp morphology (size, fusion)- Valve function (AS, AR, mixed)- Associated aortopathy or coarctation	Provides a comprehensive description of valve and aortic pathology	Designed for clinical, surgical, imaging, and research standardization

This is a single case report, and long‑term durability of the longitudinal incision approach remains to be established. Further case series and comparative studies with conventional surgical AVR and minimally invasive AVR techniques are required.

Recent studies of minimally invasive AVR often describe transverse or oblique aortotomies, such as those used in the right anterior thoracotomy “Miami Method” or transaxillary approaches [8,9]. These reports highlight the safety, reproducibility, and cosmetic advantages of minimally invasive cardiac surgery (MICS) AVR, but they do not employ a longitudinal incision. Therefore, our case represents a novel contribution, applying a longitudinal incision within a totally endoscopic setting, particularly in the context of type 0 BAV. Our experience adds to the growing body of literature suggesting that surgical strategies aimed at circularizing the annulus may offer superior outcomes.

## Conclusions

In a patient with type 0 bicuspid aortic valve and severe aortic stenosis, a totally endoscopic AVR using a longitudinal aortotomy provided direct annular visualization and enabled implantation of a large bioprosthesis with a hybrid suture strategy; early results demonstrated low transvalvular gradients, no residual aortic regurgitation (no paravalvular or transvalvular components; physiologic intra‑prosthetic washout jets may be present), and CT evidence of annular circularization, supporting the feasibility of an exposure‑driven longitudinal incision in endoscopic settings while underscoring that these findings are hypothesis‑generating and require validation regarding durability, conduction outcomes, prosthesis-patient mismatch, and comparative effectiveness versus transverse/oblique aortotomy techniques.
